# Complement‐Dominant Relapse of Mixed Autoimmune Hemolytic Anemia After Rituximab Response

**DOI:** 10.1002/jha2.70381

**Published:** 2026-08-23

**Authors:** Naru Tomigaki, Seiji Kakiuchi, Akimasa Sakamoto, Shutaro Fujioka, Isamu Harima, Hiroaki Akiyama, Ryotaro Niwa, Ikumi Takagi, Yoko Kozuki, Yoshiharu Miyata, Sou Tanaka, Hiroyuki Tsuji, Nobuko Iwata

**Affiliations:** ^1^ Department of Hematology Yodogawa Christian Hospital Osaka Japan; ^2^ Department of Artificial Intelligence and Digital Health Science Kobe University Graduate School of Medicine Kobe Japan; ^3^ Department of Clinical Laboratory Yodogawa Christian Hospital Osaka Japan

**Keywords:** clonal B‐cell population, cold agglutinin, complement, mixed autoimmune hemolytic anemia, rituximab, sutimlimab

## Abstract

**Introduction:**

Mixed autoimmune hemolytic anemia (AIHA) may show temporal shifts in its predominant effector mechanism.

**Methods:**

We serially assessed clinical course, direct antiglobulin test (DAT) specificity, cold agglutinin activity, complement markers, marrow findings, and immunofixation after rituximab response.

**Results:**

During winter relapse, the previously identified *κ*‐restricted CD20‐positive population was no longer detectable by marrow flow cytometry. Hemolysis was cold‐reactive and complement‐dominant. Hemoglobin increased without transfusion after sutimlimab, with C4 recovery and total hemolytic complement activity (CH50) suppression despite persistent cold agglutinin activity. Immunofixation later identified IgG‐*κ* and IgM‐*κ* monoclonal proteins.

**Conclusions:**

Serial reassessment may identify complement‐mediated relapse in mixed AIHA.

Trial Registration: The authors have confirmed clinical trial registration is not needed for this submission.

## 1 Introduction

Mixed autoimmune hemolytic anemia (AIHA) comprises warm‐ and cold‐reactive autoantibody components whose relative contributions to hemolysis may vary over time. Because serologic findings capture the dominant phenotype at a single time point, longitudinal reassessment may be required when relapse is not concordant with the initial classification. Rituximab targets CD20‐positive B cells, whereas sutimlimab blocks the classical complement pathway through C1s inhibition [1, 2, 3, 4]. Evidence for complement‐directed treatment in mixed AIHA remains limited.

We previously reported the initial course of this same patient, in whom warm AIHA was diagnosed at presentation and cold‐triggered relapse led to reassessment as mixed AIHA, followed by hematologic improvement after rituximab [5]. The present report describes the subsequent longitudinal follow‐up. During the following winter, complement‐dominant hemolysis recurred after the previously identified *κ*‐restricted CD20‐positive population was no longer detectable by marrow flow cytometry. Hemoglobin increased without transfusion after sutimlimab initiation. We interpreted this episode as a cold‐ and complement‐dominant relapse within mixed AIHA rather than newly developed isolated cold agglutinin disease.

## 2 Case Presentation

**FIGURE 1:**
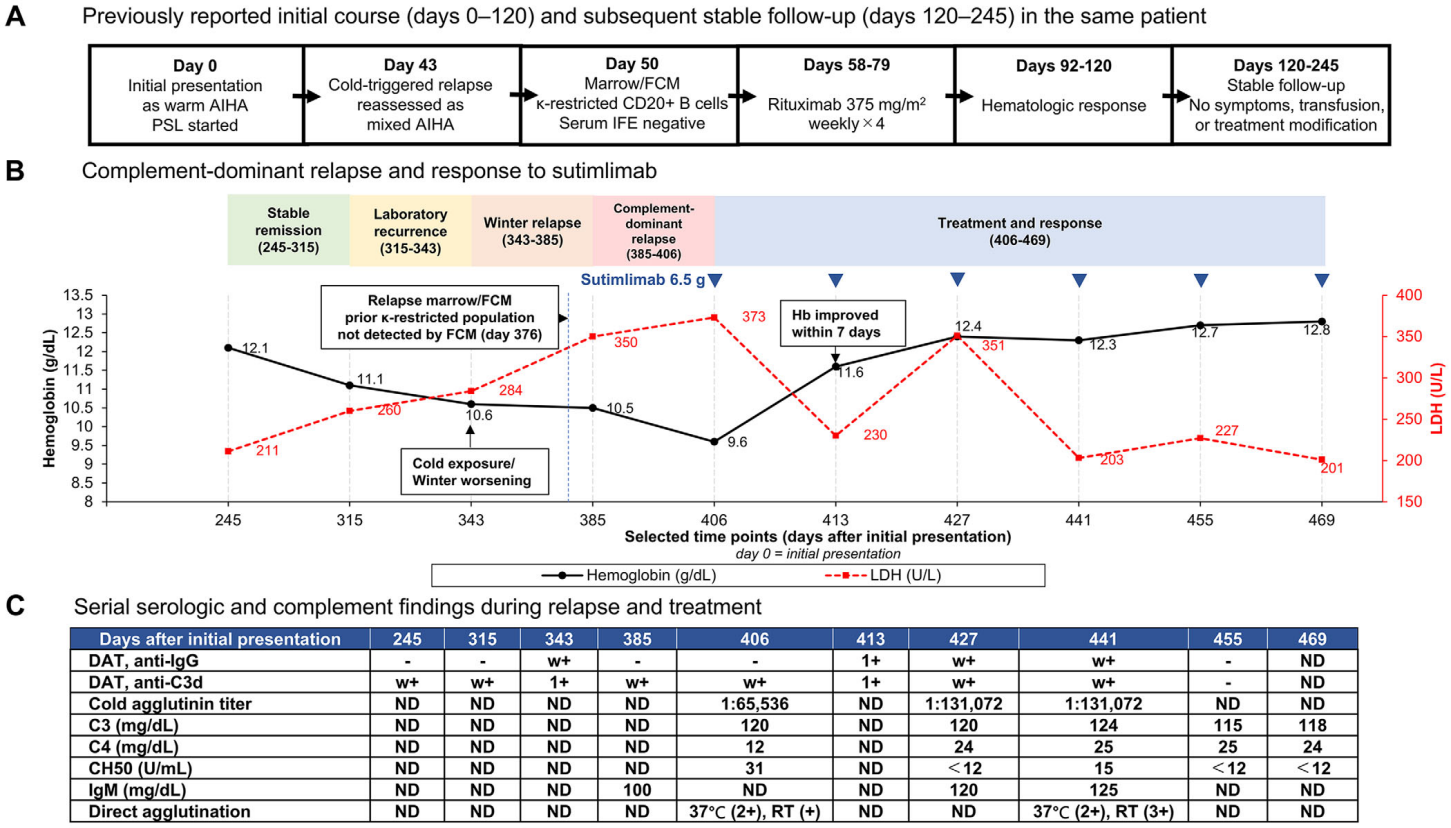
Longitudinal clinical course from initial presentation through complement‐dominant relapse and sutimlimab treatment. (A) Summary of the previously reported initial course in the same patient and the subsequent stable follow‐up. The patient initially presented with hemolytic anemia on Day 0 and was diagnosed with warm autoimmune hemolytic anemia based on direct antiglobulin test positivity for both IgG and C3d. Prednisolone was initiated. Following cold‐triggered relapse on Day 43, the markedly elevated cold agglutinin titer, cold‐reactive erythrocyte agglutination, and complement‐dominant direct antiglobulin test findings led to reassessment as mixed autoimmune hemolytic anemia. Bone marrow flow cytometry on Day 50 identified a *κ*‐restricted CD20‐positive B‐cell population, whereas serum immunofixation was negative. Rituximab 375 mg/m^2^ was administered weekly for four doses on Days 58, 65, 72, and 79. Hemolysis improved by Days 92–120, and the patient remained asymptomatic without transfusion or treatment modification through Day 245. (B) Changes in hemoglobin and lactate dehydrogenase levels at selected time points from Days 245 to 469. Laboratory evidence of recurrent hemolysis emerged on Day 315, followed by clinically evident relapse after cold exposure during the subsequent winter on Day 343. Relapse‐phase bone marrow examination and flow cytometry were performed on Day 376; the previously identified *κ*‐restricted CD20‐positive population was no longer detectable by flow cytometry. Sutimlimab 6.5 g was administered on Days 406, 413, 427, 441, 455, and 469, as indicated by the blue arrowheads. Within 7 days after the first dose, hemoglobin increased from 9.6 to 11.6 g/dL and lactate dehydrogenase decreased from 373 to 230 U/L without red blood cell transfusion. (C) Serial direct antiglobulin test results, cold agglutinin titers, complement measurements, serum IgM concentrations, and direct agglutination findings during relapse and sutimlimab treatment. Negative results are shown as “−,” whereas tests that were not determined are shown as “ND.” AIHA, autoimmune hemolytic anemia; C3, complement component 3; C4, complement component 4; CH50, total hemolytic complement activity; DAT, direct antiglobulin test; FCM, flow cytometry; Hb, hemoglobin; IFE, immunofixation electrophoresis; LDH, lactate dehydrogenase; ND, not determined; PSL, prednisolone; RT, room temperature; w+, weakly positive.

Day 0 denotes the initial presentation on January 8, 2025. The initial diagnostic course through Day 120 was reported previously, and the present report describes the subsequent longitudinal follow‐up in the same patient [5]. A woman in her 80s presented with progressive exertional dyspnea and hemolytic anemia. At presentation, hemoglobin was 8.6 g/dL and lactate dehydrogenase (LDH) was 875 U/L, with reduced haptoglobin and reticulocytosis. The direct antiglobulin test (DAT) was positive for both IgG and C3d, and no erythrocyte agglutination was observed on the peripheral blood smear. Cold agglutinin testing was not performed. Serum immunofixation and serum free light‐chain testing were not performed at presentation; both were first performed on Day 50. Computed tomography showed pulmonary emboli without lymphadenopathy, and ultrasonography identified bilateral soleal vein thrombosis. The patient was initially diagnosed with warm AIHA and received prednisolone 50 mg/day (Table 1; Figure 1A).

**TABLE 1:** Selected clinical, serologic, bone marrow, and monoclonal protein findings during the longitudinal course.

A: Clinical and serologic findings at selected time points					
Parameter	Day 0 Initial presentation	Day 43 Cold‐triggered relapse	Day 245 Stable follow‐up	Days 385–406 Pre‐sutimlimab evaluation	Day 441 During sutimlimab
Hemoglobin, g/dL	8.6	9.7	12.1	9.6	12.3
LDH, U/L	875	675	211	373	203
Total bilirubin, mg/dL	1.80	1.28	0.54	1.10	0.46
Haptoglobin, mg/dL	3	ND	27	2	23
Reticulocytes, %	10.3	ND	2.1	2.9	1.4
Peripheral blood smear	No erythrocyte agglutination	Prominent erythrocyte agglutination with occasional spherocytes	ND	ND	ND
Polyspecific DAT	1+	Weakly positive	ND	1+	Weakly positive
DAT, anti‐IgG	Weakly positive	Negative	Negative	Negative	Weakly positive
DAT, anti‐C3d	Weakly positive	Weakly positive	Weakly positive	Weakly positive	Weakly positive
Cold agglutinin titer	Not performed	1:65,536	ND	1:65,536	1:131,072
Direct agglutination	Not performed	Positive at both RT and 37°C	ND	37°C, 2+; RT, positive	37°C, 2+; RT, 3+
Serum IgM, mg/dL	153	ND	ND	100 on Day 385	125
Treatment	Prednisolone,50 mg/day initiated	Prednisolone,25 mg/day	Prednisolone,5 mg/day	Prednisolone, 5 mg/day;first sutimlimab dose (6.5 g)	Fourth sutimlimab dose (6.5 g); prednisolone reduced to 2.5 mg/day

B: Bone marrow and monoclonal protein evaluation			
Parameter	Initial evaluation (Day 50)	Relapse evaluation (Days 376–385)	Treatment‐phase serum evaluation (Days 427–441)
Bone marrow histology	No overt B‐cell lymphoma, plasma cell neoplasm, lymphoplasmacytic infiltration, or lymphoid aggregates	Normocellular marrow with relative erythroid hyperplasia; CD20‐positive B cells accounted for approximately 1% of nucleated cells, and CD138‐positive plasma cells accounted for 2%–5% of nucleated cells; no significant *κ*/*λ* imbalance by dual‐color in situ hybridization; no lymphoplasmacytic infiltration or lymphoid aggregates	Not repeated
Bone marrow flow cytometry	CD45‐gated lymphoid fraction, 14.4%; CD19‐positive cells, 45.2%; CD20‐positive cells, 44.6%; *κ*‐positive cells, 33.6%; *λ*‐positive cells, 6.4%; *κ*:*λ* ratio, 5.3:1	Previously identified CD20‐positive *κ*‐restricted population no longer detectable by flow cytometry; *κ*‐positive events, 0.8%; *λ*‐positive events, 0.4%	Not repeated
Serum protein electrophoresis	ND	ND	Suspected M‐peak measuring 0.896 g/dL on Day 427 and 0.891 g/dL on Day 441
Serum immunofixation	Negative	Not performed	IgG‐*κ* and IgM‐*κ* monoclonal proteins identified on Day 441
Serum free light chains	*κ*, 9.4 mg/L; *λ*, 8.7 mg/L; *κ*/*λ* ratio, 1.08	*κ*, 11.8 mg/L; *λ*, 10.2 mg/L; *κ*/*λ* ratio, 1.16 on day 385	*κ*, 9.9 mg/L; *λ*, 10.4 mg/L; *κ*/*λ* ratio, 0.95 on Day 441
Molecular and clonality testing	*MYD88 L265P* testing, next‐generation sequencing, and *IGH* clonality analysis were not performed at any time point.		
Imaging and systemic findings	CT showed no lymphadenopathy; subsequent CT on Day 72 showed no lymphadenopathy or splenomegaly	No palpable lymphadenopathy or hepatosplenomegaly; no fever, night sweats, or weight loss; contemporaneous CT or PET was not performed	No new systemic findings were documented

*Note*: Serum immunofixation and serum free light‐chain testing were not performed on Day 0 and were first performed on Day 50. Unless otherwise indicated, laboratory values in the Days 385–406 column were obtained on Day 406; serum IgM and serum free light chains were measured on Day 385.

Abbreviations: CT, computed tomography; DAT, direct antiglobulin test; IGH, immunoglobulin heavy chain; LDH, lactate dehydrogenase; ND, not determined; PET, positron emission tomography; RT, room temperature.

Cold‐triggered relapse occurred on Day 43 while the patient was receiving prednisolone 25 mg/day. The cold agglutinin titer was 1:65,536, anti‐IgG DAT was negative, anti‐C3d DAT remained weakly positive, and direct agglutination testing was positive at both room temperature and 37°C, prompting reassessment as mixed AIHA [5].

**FIGURE 2:**
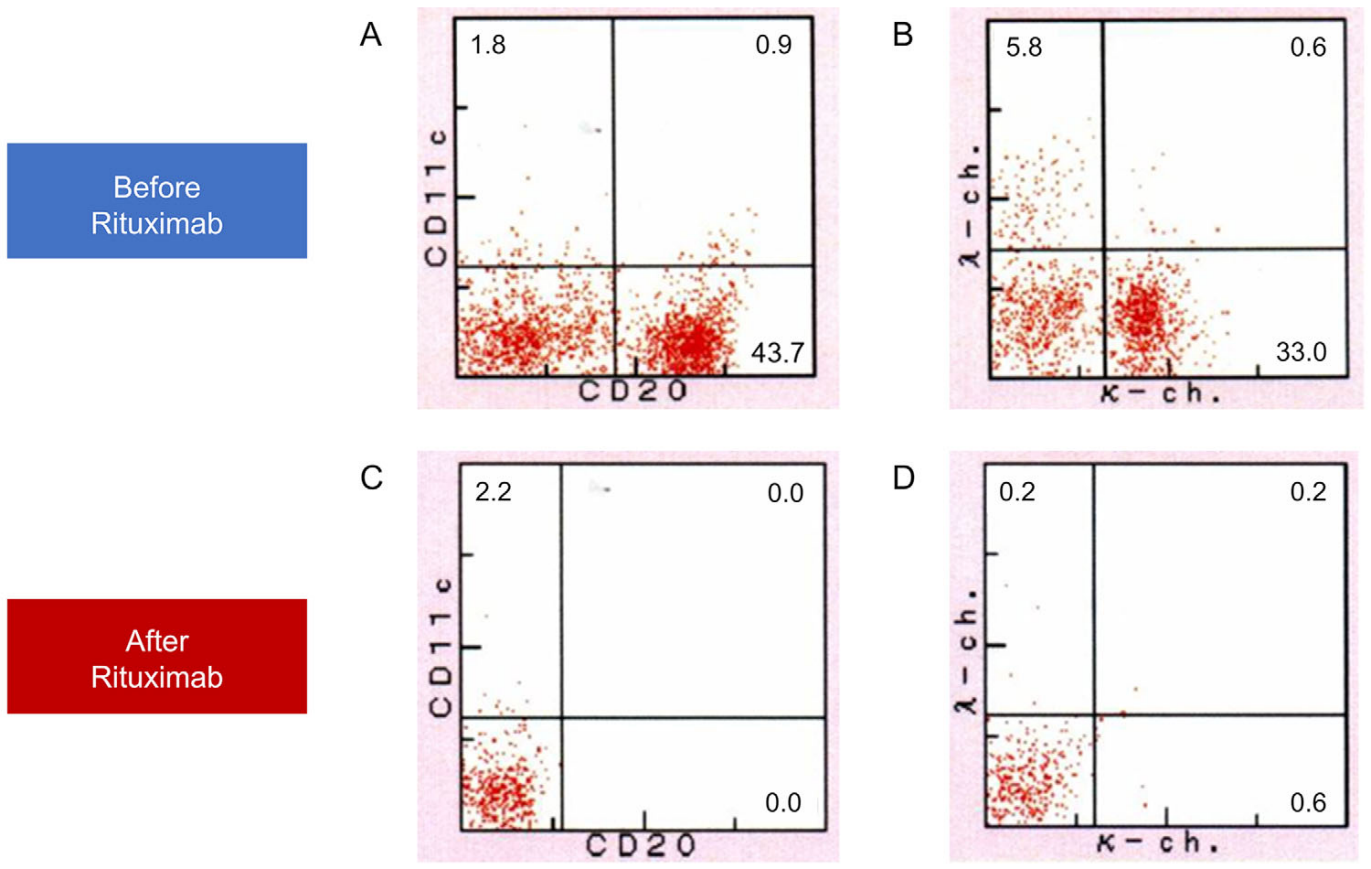
Bone marrow flow cytometry before and after rituximab. Flow cytometric analysis of bone marrow mononuclear cells gated on the lymphoid population is shown. (A, B) Before rituximab administration, a distinct CD20‐positive B‐cell population with *κ* light‐chain predominance was identified. Within the CD45‐gated lymphoid fraction, CD20‐positive cells accounted for 44.6%, and *κ*‐positive and *λ*‐positive cells accounted for 33.6% and 6.4%, respectively (*κ*:*λ* ratio, 5.3:1), consistent with a *κ*‐restricted B‐cell population. Pretreatment flow‐cytometric findings from the same patient were reported previously [5]. The panels shown here were generated from the original flow‐cytometry output. (C, D) At relapse after rituximab treatment, the previously identified CD20‐positive population was no longer detectable by flow cytometry. Only minimal *κ*‐positive and *λ*‐positive events remained (0.8% and 0.4%, respectively). These paired analyses showed that the previously detected *κ*‐restricted population was no longer identifiable by marrow flow cytometry despite subsequent winter relapse. Percentages shown within the plots are quadrant values from the original flow cytometry output, whereas the percentages reported in the text for the pretreatment specimen refer to the CD45‐gated lymphoid fraction.

Bone marrow examination on Day 50 showed no overt lymphoid or plasma cell neoplasm. Flow cytometry identified a *κ*‐restricted CD20‐positive B‐cell population, whereas serum immunofixation was negative and the serum free light‐chain ratio was normal (Table 1; Figure 2A,B). Rituximab 375 mg/m^2^ was administered on Days 58, 65, 72, and 79. Hemoglobin increased to 11.2 g/dL and LDH decreased to 267 U/L by Day 92. On Day 120, hemoglobin was 11.1 g/dL and LDH was 268 U/L. Prednisolone was tapered to 5 mg/day by Day 73.

From Day 120 through Day 245, the patient remained asymptomatic without transfusion, treatment modification, infection, or clinically relevant cold exposure. Hemoglobin remained 11.8–12.7 g/dL and LDH 211–239 U/L. On Day 245, total bilirubin was 0.54 mg/dL, haptoglobin was 27 mg/dL, reticulocytes were 2.1%, anti‐IgG DAT was negative, and anti‐C3d DAT remained weakly positive (Figure 1A,B).

Laboratory evidence of recurrent hemolysis emerged on Day 315, followed by cold‐associated exertional dyspnea and fatigue during the subsequent winter. Between Days 315 and 385, hemoglobin decreased from 11.1 to 10.5 g/dL and LDH increased from 260 to 350 U/L. DAT findings were predominantly C3d‐positive, and erythrocyte agglutination persisted (Figure 1B,C).

Relapse‐phase marrow examination on Day 376 showed erythroid hyperplasia, approximately 1% CD20‐positive B cells, and 2%–5% CD138‐positive plasma cells without significant *κ*/*λ* imbalance, lymphoplasmacytic infiltration, or lymphoid aggregates. The previously identified *κ*‐restricted CD20‐positive population was no longer detectable by flow cytometry (Table 1; Figure 2C,D). The patient had no palpable lymphadenopathy, hepatosplenomegaly, fever, night sweats, or weight loss. Contemporaneous computed tomography or positron emission tomography was not performed.

By Day 385, hemoglobin was 10.5 g/dL, LDH was 350 U/L, total bilirubin was 0.93 mg/dL, haptoglobin was 3 mg/dL, and reticulocytes were 4.1%. Anti‐IgG DAT was negative, anti‐C3d DAT was weakly positive, and serum IgM was 100 mg/dL. Serum free light chains were *κ*: 11.8 mg/L and *λ*: 10.2 mg/L, with a *κ*/*λ* ratio of 1.16. A quadrivalent meningococcal conjugate vaccine (MenACWY‐TT; MenQuadfi) and a 23‐valent pneumococcal polysaccharide vaccine (PPSV23; Pneumovax 23) were administered on Day 387 before complement inhibition.

Immediately before the first sutimlimab dose on Day 406, hemoglobin had decreased to 9.6 g/dL, and LDH had increased to 373 U/L. Total bilirubin was 1.10 mg/dL, haptoglobin was 2 mg/dL, and reticulocytes were 2.9%. The polyspecific DAT was 1+, anti‐IgG was negative, and anti‐C3d remained weakly positive. The cold agglutinin titer was 1:65,536, and direct agglutination was positive at 37°C (2+) and room temperature. Complement measurements showed C3 of 120 mg/dL, C4 of 12 mg/dL, and total hemolytic complement activity (CH50) of 31 U/mL. Prednisolone remained at 5 mg/day without dose escalation.

Sutimlimab 6.5 g was administered on Days 406, 413, 427, 441, 455, and 469. Within 7 days after the first dose, hemoglobin increased from 9.6 to 11.6 g/dL and LDH decreased from 373 to 230 U/L without red blood cell transfusion. Total bilirubin decreased to 0.48 mg/dL, haptoglobin increased to 16 mg/dL, and exertional dyspnea and fatigue improved. The polyspecific DAT, anti‐IgG DAT, and anti‐C3d DAT were each 1+ on Day 413, indicating persistence of serologic abnormalities despite the early hematologic response (Figure 1B,C).

During treatment, the cold agglutinin titer remained 1:131,072, while C4 increased and CH50 became suppressed. Serum protein electrophoresis showed suspected peaks of 0.896 g/dL on Day 427 and 0.891 g/dL on Day 441; immunofixation on Day 441 identified IgG‐*κ* and IgM‐*κ* monoclonal proteins, with a normal serum free light‐chain ratio. Direct agglutination and DAT reactivity persisted during the early hematologic response.

By Day 455, hemoglobin was 12.7 g/dL and DAT had become negative. Prednisolone was discontinued, and hemoglobin remained 12.8 g/dL with LDH 201 U/L on Day 469.

## 3 Discussion

The relapse phenotype was clinically similar to cold agglutinin disease, with winter worsening, markedly elevated cold agglutinin titers, erythrocyte agglutination, C3d‐dominant DAT findings, low C4 before treatment, and hematologic improvement after C1s inhibition. However, the same patient initially presented with anti‐IgG‐positive hemolysis and was later reassessed as having mixed AIHA after cold‐triggered relapse [5]. We therefore interpreted the later episode as a cold‐ and complement‐dominant relapse within the longitudinal course of mixed AIHA rather than as newly developed isolated cold agglutinin disease.

The rapid transfusion‐free increase in hemoglobin, reduction in LDH, recovery of C4, and suppression of CH50 after sutimlimab were consistent with classical complement pathway inhibition [3, 4]. Persistent cold agglutinin titers, direct agglutination, and DAT reactivity during the early response suggested that the treatment controlled the complement‐dependent effector phase without eliminating upstream antibody activity.

The cellular source of the antibody activity remained unresolved. The pretreatment *κ*‐restricted CD20‐positive population was no longer detectable by flow cytometry at relapse, although marrow histology showed approximately 1% CD20‐positive B cells. IgG‐*κ* and IgM‐*κ* monoclonal proteins were identified later, but their antigen specificity, pathogenicity, cellular source, and clonal relationship were not established. Detection of the IgG‐*κ* protein did not establish that the warm‐reactive erythrocyte antibody was monoclonal. Neither marrow examination showed lymphoplasmacytic infiltration or lymphoid aggregates, and pre‐rituximab CT showed no lymphadenopathy or splenomegaly. However, relapse–phase imaging, *MYD88 L265P* testing, next‐generation sequencing, *IGH* clonality analysis, and clonal tracking were not performed; an underlying small B‐cell lymphoproliferative disorder therefore could not be definitively excluded [6]. The mechanism underlying renewed anti‐IgG DAT reactivity during early sutimlimab treatment remained unresolved.

Several limitations affect interpretation of the longitudinal changes. Cold agglutinin testing was not performed at initial presentation, preventing complete characterization of the baseline phenotype. Immunofixation was not repeated between Day 50 and relapse, so the timing of the IgG‐*κ* and IgM‐*κ* proteins remains unknown. Formal thermal‐amplitude testing, *I*/*i* antigen specificity testing, *MYD88 L265P* testing, next‐generation sequencing, *IGH* clonality analysis, clonal tracking, and relapse–phase imaging were not performed. The temporal response in a single patient cannot establish the efficacy of sutimlimab across mixed AIHA.

## 4 Conclusion

The longitudinal course documented a cold‐ and complement‐dominant relapse of mixed AIHA after rituximab response. Serial reassessment identified classical complement pathway activity as the predominant effector mechanism and supported the selection of C1s inhibition in this patient.

5

N.T. collected clinical data, prepared the initial draft of the manuscript, and created the figures. S.K. conceived the report, supervised the study, critically revised the manuscript, and refined the figures. A.S., S.F., I.H., H.A., R.N., I.T., and Y.K. contributed to clinical care and data acquisition. Y.M. contributed to manuscript development and critical revision. S.T., H.T., and N.I. contributed to laboratory testing and interpretation of hematologic data. All authors approved the final version of the manuscript.

## Funding

The authors have nothing to report.

## Ethics Statement

Off‐label rituximab treatment was approved by the institutional review board of Yodogawa Christian Hospital (approval number: 2025‐007). Sutimlimab was administered within its approved indication in Japan and did not require separate institutional review board approval.

## Consent

Written informed consent was obtained from the patient for publication of this case report and accompanying images.

## Conflicts of Interest

The authors declare no conflicts of interest.

## Data Availability

The data that support the findings of this report are available from the corresponding author upon reasonable request. Patient identifiers have been removed to protect privacy.
